# Editorial: The charitable brain: the neuroscience of philanthropy and giving

**DOI:** 10.3389/fpsyg.2023.1342963

**Published:** 2024-01-04

**Authors:** Rosalba Morese, Elizabeth Elliott, Sara Palermo

**Affiliations:** ^1^Faculty of Biomedical Sciences, Faculty of Communication, Culture and Society, USI Università della Svizzera Italiana, Lugano, Switzerland; ^2^Centre for Clinical Brain Sciences, University of Edinburgh, Edinburgh, United Kingdom; ^3^Department of Psychology, University of Turin, Turin, Italy; ^4^Neuroradiology Unit, Diagnostic and Technology Department, Fondazione Istituto di Ricovero e Cura a Carattere Scientifico (IRCCS) Istituto Neurologico Carlo Besta, Milan, Italy

**Keywords:** psychology of giving, giving behavior, prosociality, altruism, empathy, social cognition, social dynamics, metacognition

The “social brain” refers to the idea that the human brain has evolved to be particularly adapted for social interactions and relationships. It encompasses various cognitive and neural mechanisms that enable humans to understand, navigate, and thrive in social environments (Morese and Palermo, 2022). Key components of the social brain include theory of mind (the ability to understand and infer the mental states of others), empathy (the ability to understand and share the feelings of others), and social cognition (the capacity to process and interpret social information) (Morese et al., 2018; Morese and Palermo, 2022). The social brain has evolved because humans are inherently social animals, and the ability to cooperate and collaborate with others has been a significant evolutionary advantage.

Altruism can be seen as an expression of the social brain, as it relies on an individual's capacity for empathy, compassion, and the ability to recognize the needs of others. Altruistic behaviors are often driven by a desire to promote the wellbeing of the broader community. Philanthropy is an expression of altruism in a more organized and often structured manner. It can encompass a wide range of activities, from donating to non-profit organizations to founding one's charitable foundations.

The Research Topic provides a first overview of the knowledge surrounding the multiple ways in which daily life is interwoven with prosocial behaviors. The Research Topic comprises 4 contributions represented by 3 original research articles and 1 opinion paper. To summarize, the editorial contributions published in the Research Topic represent an important first step to open a multidisciplinary discussion on a topic that nowadays has increasingly greater implications for the wellbeing of individuals and communities.

The social brain is particularly relevant during adolescence, as the brain continues to develop, and individuals become more attuned to social and interpersonal dynamics (Sipes et al.). The social brain, prosociality, and compassion are interconnected in the context of adolescent development. The ongoing maturation of the brain, moral development, and peer interactions all contribute to adolescents' increasing capacity for and engagement in prosocial and compassionate behaviors, as they become more attuned to the needs and emotions of others. In innovative research, the Authors investigate a recent model: the domain-general development network (Do-GooD) highlighted in the context of social cognition and neurophysiological aspects related to compassion and prosocial behavior (Sipes et al.). This novel study provides important insight into the neurodevelopmental basis of prosocial cognition during the formative stage of adolescence.

In an opinion paper, conscious capitalism is read and interpreted through the lens of neuroscience (Palermo). The connection between the social brain and conscious capitalism lies in the idea that our inherent social nature, empathy, and concern for the wellbeing of others can influence the way businesses and organizations are structured and how they operate. Conscious capitalism recognizes that businesses can benefit from aligning their values and actions with the social consciousness and ethical values that the social brain is thought to underpin. In this sense, conscious capitalism can be seen as an approach that acknowledges and harnesses elements of the social brain to create businesses with a greater societal impact and a sense of purpose beyond profit (Palermo). Altruistic behavior increases happiness in individuals and organizations. In a final original article, the Authors investigated this phenomenon across cultures, distinguishing between different kind of cultures based on cultural norms, such as individualistic and collectivist (Weiss-Sidi and Riemer). They first measured dispositions based on different cultural aspects. Then they investigated the important role of cultural orientation on the impact of the use of monetary units related to self and other people. Finally, they study the altruism-happiness association with altruistic behavior in the comparison process of individualistic and collectivist cultures in which interdependence and altruistic behaviors are inherent (Weiss-Sidi and Riemer). The authors propose that individualistic cultures are associated with a more “impure” or self-focussed form of altruism in contrast to a “pure” other-focussed altruism amongst collectivists.

Linked to these suggestions is research on “corporate philanthropy–corporate financial performance” (Chen and Bu). Although numerous studies have been conducted on “the relationship between corporate philanthropy and corporate financial performance (CFP), theoretical analysis focusing on the legitimacy-based mechanism and the moderating role of key executives' psychological characteristics is scarce” (Chen and Bu, p. 1). “Hometown attachment is a special form of place attachment in environmental psychology, people's psychological attachment to their hometown and the state of maintaining an intimate emotional connection with it” (Chen and Bu, p. 1). The study proposed by Chen and Bu draws the Chinese cultural origin in the setting of legitimacy of donations.

In summary, social brain provides cognitive and emotional foundation for human social interactions, including the capacity for empathy and altruism. Altruism is the selfless, individual-level expression of social cooperation and compassion, while philanthropy represents a more organized and often institutionalized form of altruism on a larger scale. These concepts are interconnected through the fundamental human drive to support and benefit others, and they all play a role in shaping our social and cooperative behavior (Auriemma et al., 2020; Morese et al., 2020; Morese and Longobardi, 2022).

## Author contributions

SP: Writing—original draft. RM: Writing—review & editing. EE: Writing—review & editing.
